# Entangling Reciprocity With the Relational in Narrative Inquiry

**DOI:** 10.1177/10778004231172227

**Published:** 2023-06-05

**Authors:** Bodil H. Blix, Jean Clandinin, Pamela Steeves, Vera Caine

**Affiliations:** 1 UiT The Arctic University of Norway, Tromso, Norway; 2 Univerisity of Alberta, Edmonton, Canada; 3 Univerisity of Victoria, British Columbia, Canada

**Keywords:** narrative, methods of inquiry, reciprocity, relational ontology

## Abstract

In this article, we develop, through drawing forward fragments of our experiences, a concept of reciprocity as always situated within the relational ontology of narrative inquiry. Reciprocity is most commonly understood within a transactional sense, an exchange of goods. We show important aspects of reciprocity in narrative inquiry, including the importance of intentionally creating and responding to spaces where reciprocity occurs and can be sustained over time and place, and the potential reciprocity holds to change who we, and those with whom we work, are. As we reconsider the ways in which reciprocity is not understood as a transaction in a relational methodology, new questions about the entanglement of reciprocity and recognition emerge. We understand that recognition does not necessarily have to be reciprocal, but recognition is necessary to compose a space where reciprocity can live in our ordinary interactions with others.

## Introduction

The arrangement of Ghalia’s table called up my [Vera’s] memories of home in Germany and the time my mother spent setting the table for friends. She always paid attention to details, a gesture of care and attentiveness. As I watched Ghalia that evening, I could see parts of my mother in her. My feelings of being unsettled and of meeting my mother that evening were not ones I had anticipated. The plates and glasses were carefully arranged, as was the sequence of food placed at the center of the table and, eventually, on my plate. Ghalia smiled as she watched me, while her children and husband watched her. As I watched Ghalia’s children’s and husband’s eyes follow her that evening, I saw joy in their eyes. It was as if they were meeting their mother and wife again.^1^

In Vera’s narrative account of her experience at a dinner with Ghalia and her family, who had come to Canada from Syria as refugees, we wondered if, in this moment, worlds were shifting as lives came together in a spirit of reciprocity. Thinking with Vera’s experience, we wonder: What do we mean by a spirit of reciprocity? How are we, both researchers and participants, changed through the reciprocal relationships we compose in narrative inquiry studies? These questions, we recognize, are linked to complexities that interweave reciprocity with the relational in narrative inquiry.

Our questions prompted our return to earlier writings, including a chapter written by Clandinin and Caine (2013) where they laid out touchstones for judging the quality of narrative inquiries and describing narrative inquiry spaces as “spaces of belonging for both researchers and participants; spaces that are marked always by ethics and attitudes of openness, mutual vulnerability, reciprocity, and care” (p. 169). Later, Clandinin and colleagues (2018) wrote,

Such a relational ontology requires that we undertake research with an understanding of relational ethics that call us to larger questions of who we are in relation with participants but also who we are in relation with the larger world or worlds that people, including us as researchers, inhabit. This relational ontology interwoven necessarily with a relational ethics calls us to consider mutuality, respect, and reciprocity. But it also calls us to questions of responsibility to the person and to the worlds in which we are nested, to questions of complicity in the worlds within which we currently exist as well as to future worlds that our work leads into. (p. 20)

While we connected reciprocity to the relational ontology of narrative inquiry, we did so without exploring the ways reciprocity was central to what it meant to work and live within a relational ontology. As we lingered with this lacunae in our texts, we began to wonder if our silence about reciprocity allowed dominant narratives of research to fill in these gaps and silences without our explicit resistance to the more taken-for-granted meanings of reciprocity as exchanges for mutual benefits. We returned to the work of Noddings (1984) within an ethic of care and noted her attention to reciprocity within care as distinct from “that of ‘contract theorists’” (p. 4). She sees reciprocity as “the most important problem” (p. 4) in understanding an ethic of care.

As we turned to the research of others, we noted reciprocity was most frequently seen as something transactional, a kind of interaction between researcher and participants that forefronted issues of power and obligations (Tubaro, 2021; von Vacano, 2019). We observed that reciprocity is often framed as “user/patient involvement,” “participant/community engagement,” “‘paying’ for people’s time,” or as “giving voice.” We wondered about our lack of attention to developing a more explicit concept of reciprocity in narrative inquiry, with attention to the importance of engaging in the relational through coming alongside participants with an openness to uncertainty (Dewey, 1929), to be perplexed (Addams, 1902), to be astonished (Minnich, 2014), and to change (Connelly & Clandinin, 1990). We wondered if our silences had allowed the development of transactional understandings of reciprocity for those engaged in various approaches to research including some narrative inquiry studies.

In this article, we develop, through drawing forward fragments of our experiences, a concept of reciprocity as always situated within relational ontologies. We show the importance of intentionally creating spaces where reciprocity occurs. We understand that these intentional spaces for reciprocity are spaces of co-creation, sometimes opened by researchers, sometimes by participants. These co-created spaces are those where reciprocity does not end but can be sustained over time. Furthermore, we show that reciprocity changes who we, and those who work with us, become through our engagement with each other; it requires a willingness to enter into such spaces with openness to becoming otherwise (Greene, 1995), to change the stories we live and tell.

## Our Understanding of Narrative Inquiry

Narrative inquiry, as both a qualitative research methodology and a way of viewing the phenomena under study, was developed by Connelly and Clandinin (1990) and Clandinin and Connelly (2000). They wrote,

People shape their daily lives by stories of who they and others are and as they interpret their past in terms of these stories. Story, in the current idiom, is a portal through which a person enters the world and by which their experience of the world is interpreted and made personally meaningful. Narrative inquiry, the study of experience as story, then, is first and foremost a way of thinking about experience. Narrative inquiry as a methodology entails a view of the phenomenon. To use narrative inquiry methodology is to adopt a particular view of experience as the phenomenon under study. (Connelly & Clandinin, 2006, p. 375)

They built on the pragmatist ideas of Dewey (1938) with their focus on experience within a metaphorical three-dimensional space composed of sociality, temporality, and place. Each inquiry is marked by relational ethics (Clandinin et al., 2018), which makes visible spaces between the researcher and participants and, in so doing, highlights social responsibilities and actions. It is important to recognize that the narrative inquiry shapes the phenomenon under study, the methodology to study experience (Clandinin, 2013), as well as the lives of those involved, which makes it significantly different from other narrative research approaches in which the narrative is considered “the object for careful study” (Riessman, 2008, p. 14).

A narrative inquiry proceeds from an ontological position, a curiosity about how people are living and the constituents of their experience; narrative inquirers seek to understand and evoke experiences from within an inquiry (Clandinin & Rosiek, 2007). As much as narrative inquiry arises from puzzles around people’s experience, it involves an ontological commitment, as well as an understanding of inquiry as a negotiated research practice. Thinking about reciprocity in narrative inquiry, we turned our attention to Jane Addams, who connects the ideas of reciprocity with social ethics; as Višňovský (2011) points out, Addams “had even spoken explicitly about the ‘principle of reciprocity’ as a substantial part of morality and social relations altogether. According to her, none of them is possible without reciprocity; that is, neither ethics nor social life” (p. 441). Following Addams, and our emerging narrative understandings of reciprocity, reciprocity is more than the exchange of things, or the privileges granted in exchange of something.

As Clandinin and Rosiek (2007) noted, a narrative ontology precedes the emergence of research puzzles and calls forth obligations and commitments in narrative inquiry. A narrative ontology calls upon researchers to enter into what Dewey (1934) termed ordinary experience, which we refer to as “the practice and artistry of lives lived” (Caine et al., 2013, p. 576). Within a relational ontology, experiences are continuously interactive, resulting in changes in both people and the contexts in which they interact (Dewey, 1938); people’s lives are composed and re-composed in relation with others who are also living storied lives. As Clandinin and Connelly (2000) wrote, no one walks away from a narrative inquiry unchanged. This understanding opens into the importance of reciprocity being more complex and nuanced than what a transactional view implies.

As we turn toward Vera’s experience and what we imagine as Ghalia’s experience, questions around the meaning of reciprocity become visible. As Ghalia and her family welcomed Vera into their home and shared in the ordinary pastime of eating food together over several months, Vera awakened to wonders about who she had become in Ghalia’s life and in the life of Ghalia’s family. As she attended to these wonders, Vera was beginning to ask questions of an ethical nature, questions in which she wondered about reciprocity, with its link to relational ethics, in narrative inquiry.

## Returning to Experience: A View Toward Reciprocity in the Relational

We found that often when we spent time with the participating families, they provided familiar food from their home countries for us. In a way, we could see this as participants offering us gifts as a way of expressing their regard for us, a way of inviting us into their lives. We saw that the way families shared food with us was marked by what we named as a spirit of reciprocity.

Ghalia carefully placed some food on my plate and then served her family. The smells of the food were unfamiliar to me and I did not recognise the dishes Ghalia had prepared. Ghalia’s oldest boy told me that they had not eaten some of the dishes their mother was serving since they left Syria. I thought I could see in his eyes how much he missed these once familiar dishes. As she served me and then her family, I wondered what our relationship meant to Ghalia, and what it meant to me. I sensed that I had become important in Ghalia’s life and the life of her family; I recognized that I cared about Ghalia, that I felt a connection with her. I was deeply touched by Ghalia’s gesture of preparing special dishes and for allowing me to metaphorically travel with her to Syria, the country of her birth.

The food dishes Ghalia prepared for this meal were ones Ghalia had not prepared since the family arrived in Canada. Her husband saw it as an expression of the importance of Vera’s visits with the family, a kind of marking of a special relationship that was growing between Vera and his family. Ghalia’s son made visible his longing for his mother’s food and how much it meant to him to experience his mother’s agency in her willingness to prepare the special dishes again now in this new place. We felt a sense of her son’s desire in the words of Nayeri (2019), “[w]hat rivers of memory flow quietly in the veins, waiting for a note, an image, a smell so that they can gush up to the surface” (p.164). Perhaps his memories of his family and his mother before their lives had changed so dramatically gushed to the surface as he experienced the smells and sights of the food prepared by his mother after so long away from her home country. But perhaps what Vera saw in the son’s eyes was not only his appreciation of the mother he once knew and the acknowledgment of his mother’s agency but also a recognition of Vera’s appreciation of Ghalia’s skillfulness. As Banerjee (2022) points out, this resonates with Jane Addams’ story of how Angelina, the Italian girl, “came to respect her mother through witnessing other people’s admiration of her mother’s spinning skills in the Labor Museum” (np).

Ghalia’s husband must have sensed my concern over the time Ghalia spent to prepare this meal, as he explained to me how much my visits meant to his family. He explained that his wife had not cooked these dishes for a long time; that life in this new place had not been easy for them as a family and that it had exacerbated Ghalia’s mental health issues. I remember looking at Ghalia in that moment, wondering if I was the reason she prepared this meal.

Both Vera’s and Addams’ stories are reminders that relationships in narrative inquiry are multiple and complex and that they change not only researchers and participants but also people close to them. As such, through her cooking, Ghalia created an intentional space where reciprocity could occur and flourish, not only between Vera and herself but also in her family and between Vera and Ghalia’s family. When Vera accepted the invitation to dinner at Ghalia’s home, she was unaware that Ghalia would see it as a moment in which she could express her sense of herself in a relationship with Vera. Vera’s anticipated presence in Ghalia’s home allowed Ghalia to intentionally open a space for Vera, hoping Vera would take up her invitation. Vera was surprised to find herself within a space of reciprocity that Ghalia had created. Although initially perplexed by what was happening, Vera saw this as a possibility for a different relationship, one that signaled the possibility of change for both her and for Ghalia. Intentional creation of spaces for the expression of reciprocity is a part of the relational ontology of narrative inquiry.

## Returning to Experience: Creating Spaces for Reciprocity

Helena, an older woman from the countryside, was one of the residents in the sheltered ward for persons living with dementia.^2^ Helena struggled to understand why she was in the ward, and she spent the days wandering back and forth between the locked doors at each end of the long corridor. I [Bodil] could see how Helena’s frustration and stress built up throughout the days because she felt she had to get out of there to get home to her small children who needed her. I remember watching her, thinking that her situation reminded me of the exhausting nightmares I experienced every now and then, in which I desperately had to go somewhere but was never able to get there. At first, I tried comforting Helena by telling her that her children were grown up and managing fine without her. But nothing I said would make her relax. And I felt unsure about the effect of the medications Helena was put on to relieve her stress and restlessness.

Narrative inquiry is a turn toward experience, and as narrative inquirers, we are called to attend to the worlds in which people live their lives. We see in Bodil’s narrative account, two women, living in two different worlds (Lugones, 1987). In the world Helena was living, she was the mother of young children who needed her care, and in the world Bodil was living, Helena was a person living with dementia, a person who needed care. As a young and inexperienced nursing student, Bodil instinctively tried to welcome Helena into *her* world by comforting Helena by referring to “facts” based in her own world (*your children are grown up and managing fine without you*), only to experience that Helena’s frustration and anxiety was growing. The situation was marked by Addams’ understanding of perplexity, that is, “a situation that baffles and confuses her, because her usual understandings and responses are inadequate to explain or transform [the] situation” (Seigfried, 2002, p. xxv). However, Helena’s embodied experience of being trapped in the ward, unable to get home to her children, resonated with Bodil’s embodied experiences of exhausting nightmares about not being able to get to places she needed to be. Embodied experiences shape our opportunities to travel to each other’s worlds. Perhaps Bodil’s recognition of the experience of being trapped was an opening for her to travel to Helena’s world, rather than trying to make Helena travel to Bodil’s world. Lugones (1987) described “world” traveling as “a way of identifying with [the other] because by traveling to [the other’s] world we can understand what it is to be them and what it is to be ourselves in their eyes” (p.17). And perhaps, through this “world” traveling, a space was created that Helena and Bodil could commonly explore.

I had noticed that there was a spinning wheel and a basket with wool in Helena’s room. One day I asked her if she could teach me how to use it. The following weeks, Helena and I spent much of our time together by the spinning wheel. Day after day, Helena demonstrated how she used the spinning wheel, and then patiently watched me trying to copy her. I never became particularly good at spinning. However, the spinning did something to our relationship. While spinning, Helena was the expert, and became less anxious. While spinning, both Helena and I experienced that I needed her more than she needed me. And I also saw something new while sitting with Helena by the spinning wheel—a woman with skills and knowledge, rather than a patient with dementia.

Addams (1902) believed that “action is indeed the sole medium of expression for ethics [. . .] that a situation does not really become moral until we are confronted with the question of what shall be done in a concrete case and are obliged to act upon our theory” (p. 273). We realize, while thinking with Bodil’s experience alongside Helena, that the fruitless attempts at comforting Helena by stating that her children were grown up and managed fine without her, were Bodil’s attempts “to act.” However, her “actions” only told Helena that she was not needed. It was through the domestic details embodied in the spinning that a unique space between Helena and Bodil was created, a space open to different relations, a space in which both could be, and become, otherwise. While initially Bodil was caught in her own world, assuming Helena could understand Bodil’s world, the spinning offered the possibility of a space in which Bodil gave up her power and entered Helena’s world. The “ethos of reciprocity offers a radical alternative to the framework of unequal power and resources. The ethos of reciprocity frees us from the dilemma of “for whose good?” (Charon, 2014, p. S23). Sitting with Bodil by the spinning wheel, Helena was able to live a narrative about herself forefronting her experiential knowledge, competencies, and capacity to care. To intentionally compose a space for this new narrative to unfold, Bodil had to let go of the safety of the stories she knew, the dominant narratives about people living with dementia. By the spinning wheel, Helena and Bodil collaboratively composed a space in which both Helena and Bodil could engage in the reciprocal act of care because “the ethos of reciprocity grounds care in a respectful generosity in which neither the giver nor the receiver is diminished by the gift” (Charon, 2014, p. S21). Noddings (2013) noted,

[t]he reciprocity in caring relations is not contractual; that is, we do not expect the cared-for to balance the relation by doing what the one-caring (or carer) does. [. . .] The world is not divided into carers and cared-fors as separate and permanent classes. We are all inevitably cared-fors at many times and, ideally, most of us are carers. (p. xxi)

However, a common space, or “world,” must be composed in which such reciprocal caring relationships can grow.

## Returning to Experience: Reciprocity Over Time and Place

Jette and I [Pamela] first met in a graduate class and I was very aware of our different positions. Jette was a highly regarded school principal. I was a part-time teacher and teacher-librarian. It was our shared love of children’s literature and the ways it was important to adults as well as children that brought us together. The stories and illustrations ignited our imaginations about different life experiences. We began to meet on a regular basis. We often raced down to the library to look up the etymology of words we were wondering about, words like company, companionship. How might a change of companionship shape the journey in a school? We liked to play with ideas from our conversations, and in doing so we were also sharing stories of our experiences.^3^

Considering reciprocity in a caring relation, Noddings (1984) draws on Buber’s (1937/2003) ideas from his book *I and Thou* and links reciprocity to recognition. She emphasizes that caring is completed in the encounter with the other. Through their conversations, Jette and Pamela recognized each other in their mutual feelings about children’s literature.

After a year of graduate courses, Jette needed to leave to take up a principal assignment at a new school. Jette was nervous about going to a new school. I felt her anxiety as I too had experienced times and places where I was not known. Now Jette was entering such a time and place, a transition for her. We began to imagine how we might continue our book/life conversations. Maybe we could think together about transitions. Our relationship allowed me to think more about who we each were and who we were to each other. I wondered: Could we find ways to continue our conversations within my doctoral work? Eventually, in conversation with Jette, a narrative inquiry evolved that was meaningful to both of us, a narrative inquiry into the experience of composing identity in transition. (Steeves, 2000)

Their first recognition of each other was followed by the shared creation of an intentional research space, an expression of reciprocity within a caring relationship. P recalled how Noddings (1984) drew attention to the words of Buber that “relation is reciprocity” (p. 74). In the narrative inquiry at Jette’s school, P felt a deeper sense of who she was, and who Jette was, as they listened and responded to each other. Their stories to live by (Connelly & Clandinin, 1990) were shifting through their curiosity and their work together marked by reciprocity.

Conversations in Jette’s school office continued over the next few years and led to other studies. Eventually, I moved to another city.

Relationships characterized by reciprocity and dialog are created where trust and understanding are present, often developed within long-standing relationships and time spent together. The narrative inquiries Pamela and Jette participated in kept open a space to continue the relationship first realized in the sharing of children’s literature and continued through several research endeavors.

Off and on Jette and I kept in touch. But during the pandemic we began, and now often, engage in skype conversations every couple of weeks. We talk about many things. Often there is the phrase “and you and I talked about this” taking us back in time and propelling us forward in retellings. For several years we’ve explored our own family stories, ancient and more recent. We share our thoughts and writing of past and present experiences along with wonders around the future. And who knows, maybe someday we will co-author a children’s book.

Pamela and Jette’s relationship has stretched over many years. It attests to the reciprocity nurtured in the space of narrative inquiry. Noddings (2013) reminds us that “[t]he attempt to maintain a caring relation is an attempt to keep the doors of communication open [. . . and it] requires continuous reflection on the part of the carers” (xvii). The continued intention to keep the space open made visible the reciprocity embodied in their relationship. More recently, Jette and Pamela’s skype conversations triggered memories of conversations from long ago places. Being able to pick up threads of conversation over the years reflected a back and forthing to connect and reconnect their relationship; it was another marker of reciprocity.

## Conceptualizing Reciprocity in Narrative Inquiry: Opening Questions of Recognition

By drawing forward fragments of our experiences from our research and practice, we showed important aspects of reciprocity in narrative inquiry, including the importance of intentionally creating and responding to spaces where reciprocity occurs and can be sustained over time and place and the potential reciprocity holds to change who we, and those with whom we work, are.

Reciprocity is situated “always in the midst of stories,” our own stories, those of participants, and the stories we co-compose. Engaging in narrative inquiry work calls forward an intentional focus on reciprocity, whereby reciprocity cannot be demanded but must be invited through the creation of spaces for reciprocity. We are called to open spaces and respond to spaces that, as Addams (1902) writes, embody an ethics in the action we take, particularly when we are faced with perplexing situations, when we are not sure, when we are unsettled, and when we are called to make visible who we are (Dodd et al., 2022).

The transforming of researchers’ lives through reciprocity holds the potential, whether we are awake to it or not, of shaping our research puzzles. It shapes what we are curious about. Inherent in our understanding of narrative inquiry is the acknowledgment that the phenomenon under study, the participants, and the inquirer are all changing throughout the inquiry process—narrative inquiry is a reciprocally, ontologically transforming process. We enter relationships with an openness and willingness to be changed. It cannot be otherwise if reciprocity centers our work.

As we gathered threads across our experiences, we returned to considerations of recognition and the part that recognition plays in creating spaces that make reciprocity possible. As we unpacked our stories of experience and reconsidered the ways in which reciprocity was not understood as a transaction in a relational methodology, new questions about the entanglement of reciprocity and recognition began to emerge. We were perplexed by how frequently we assumed recognition as central to reciprocity but did not linger to consider just what we meant by recognition.

We came to understand that recognition does not necessarily have to be reciprocal, but recognition is necessary to compose a space where reciprocity can live in our ordinary interactions with others. Spaces composed as a response to recognition can be spaces of reciprocity. It was Ghalia’s recognition of Vera that led her to invite Vera to a dinner comprised of foods she cooked from her home land of Syria. *I remember looking at Ghalia in that moment, wondering if I was the reason she prepared this meal.* Ghalia acts upon her recognition of Vera by inviting her to dinner and, in so doing, metaphorically invite her to travel to her home country through serving one’s familiar dishes. In Ghalia’s intentional act, she invites Vera to a space of reciprocity, which calls Vera to remember her mother. In this moment of eating the dinner and being reminded of her mother, Vera recognizes what Ghalia is doing through creating this dinner. Without Ghalia’s recognition of Vera, and Ghalia’s intentional invitation, reciprocity would have not been possible.

Bodil initially failed to recognize Helena, even though she desired to do so. Bodil was open to being perplexed (Addams, 1902) following her first instinctive and fruitless attempts to ease Helena’s anxiety. In a moment of recognition, Bodil saw her own embodied experiences in the situation Helena was living. “*I remember watching her, thinking that her situation reminded me of the exhausting nightmares I experienced every now and then, in which I desperately had to go somewhere but was never able to get there*.” Following Bodil’s experience of recognizing a similar experience in her nightmares, she was able to travel to Helena’s world with loving perception (Lugones, 1987), to create a space for reciprocity, a space in which Helena was the teacher and Bodil was the learner.

For Pamela and Jette, the initial recognition was around their shared love of children’s literature and the possibility of imagining otherwise (Greene, 1995). But the shared space of reciprocity was sustained by their recognition of how schools might be more inclusive as communities of learning and Pamela’s recognition of their shared fears around transitions. *I felt her anxiety as I too had experienced times and places where I was not known.* The invitation to a space of reciprocity within Pamela’s doctoral work and Jette’s principalship in a new school reflected their shared fear. We see the sustenance of spaces for reciprocity over time in Pamela’s and Jette’s work over many years—in connecting over children’s literature, to a shared interest and experience of transitions, and then shifting to Jette’s interest in the experiences of student teachers and learning to teach.

What we have learned through our development of a concept of reciprocity within the relational ontology of narrative inquiry is the central process of being able to recognize the other, to being able to engage in perhaps what Lugones names world traveling with loving perception rather than arrogant perception (Lugones, 1987). Lugones’ concept of loving perception within world traveling is perhaps akin to what we intend by recognition that opens the intentional space of reciprocity. The entanglements of reciprocity within a relational ontology are inextricably linked to recognition.

## Notes

1. In 2018, Vera and Jean, alongside other researchers (Kubota et al., 2021), started to work with 11 Syrian refugee families with preschool children in a narrative inquiry. The study purpose, motivated by an interest in the lives of refugees, is to understand the experiences of refugee children and their families, as well as the experiences of teachers as their lives met in early childhood settings in Canada. The study’s intent was to offer insights into questions of identity-making and agency, particularly framed within what it means to experience social inclusion and belonging. They met with families within 2 years of their arrival in Canada. As members of the research team, we listened to their stories of experience and came alongside them as they negotiated their lives in new places. We were particularly interested in the lives of young children and their families as they made sense of unfamiliar institutional settings, including schooling.

2. Almost 30 years ago, as a young nursing student, Bodil did a practicum in a sheltered ward for persons living with dementia. She entered this ward with very little experience of coming alongside people living with dementia and with the limited knowledge about dementia that she had accessed through her nursing school text books.

3. Following the completion of Pamela’s doctoral work (Steeves, 2000) with Jette as a participant, she continued her visits with Jette. Alongside others, Pamela and Jette explored the stories of diverse individual children (Clandinin et al., 2006). Later, they participated in a narrative inquiry with student teachers. Their puzzle was around the experiences of student teachers when their practicum placements remained at the same school (McKenzie-Robblee & Steeves, 2008). After the completion of the study, Pamela continued weekly meetings with Jette even after Jette had left the school district and Pamela had moved to another city.

## References

[bibr1-10778004231172227] Addams J. (1902). Democracy and social ethics. McMillan.

[bibr2-10778004231172227] Banerjee A. (2022). Dialogue, liminality, and a spatial ethic of reciprocity in difference: Jane Addams’s social ethics at the confluence of feminism and pragmatism. In Shields P. M. Hamington M. Soeters J. (Eds.), The Oxford handbook of Jane Addams (online ed.). 10.1093/oxfordhb/9780197544518.013.39

[bibr3-10778004231172227] Buber M. (1937/2003). I and Thou ( Smith R.G. , Trans.). T & T Clark. (Original work published 1937)

[bibr4-10778004231172227] Charon R. (2014). Narrative reciprocity. Hastings Center Report, 44(s1), S21–S24. 10.1002/hast.264 24408702

[bibr5-10778004231172227] Caine V. Estefan A. Clandinin D. J . (2013). A return to methodological commitment: Reflections on narrative inquiry. Scandinavian Journal of Educational Research, 57(6), 574–586. 10.1080/00313831.2013.798833

[bibr6-10778004231172227] Clandinin D. J . (2013). Engaging in narrative inquiry. Left Coast Press.

[bibr7-10778004231172227] Clandinin D. J. Caine V . (2013). Narrative inquiry. In Trainor A. Graue E . (Eds.), Reviewing qualitative research in the social sciences (pp. 166–179). Taylor and Francis/Routledge.

[bibr8-10778004231172227] Clandinin D. J. Caine V Lessard S . (2018). The relational ethics of narrative inquiry. Routledge.

[bibr9-10778004231172227] Clandinin D. J. Connelly F.M . (2000). Narrative inquiry: Experience and story in qualitative research. Jossey-Bass.

[bibr10-10778004231172227] Clandinin D. J. Huber J. Huber M. Murphy M. S. Murray Orr A. Pearce M. Steeves P. (2006). Composing diverse identities: Narrative inquiries into the interwoven lives of children and teachers. New York: Routledge.

[bibr11-10778004231172227] Clandinin D. J. Rosiek J. (2007). Mapping a landscape of narrative inquiry: Borderland spaces and tensions. In Clandinin D. J. (ed.). Handbook of narrative inquiry: Mapping a methodology (pp. 35–75). SAGE.

[bibr12-10778004231172227] Connelly M. Clandinin D. J . (1990). Stories of experience and narrative inquiry. Educational Researcher, 19(5), 2–14.

[bibr13-10778004231172227] Connelly F. M. Clandinin D. J . (2006). Narrative inquiry. In Green J. Camilli G. Elmore P . (Eds.), Handbook of complementary methods in education research (pp. 375–385). Lawrence Erlbaum.

[bibr14-10778004231172227] Dewey J. (1929). The quest for certainty: A study of the relation between knowledge and action. Minton, Balch & Company.

[bibr15-10778004231172227] Dewey J. (1934). Art as experience. Capricorn Books.

[bibr16-10778004231172227] Dewey J. (1938). Experience and education. Collier Books.

[bibr17-10778004231172227] Dodd J. Lessard S. Caine V. Toogood K. Clandinin J. (2022). Thinking with perplexities in the context of social inclusion, refugees, and schools: Methodological learnings. Qualitative Inquiry, 29(5), 551–557. 10.1177/10778004221122360

[bibr18-10778004231172227] Greene M. (1995). Releasing the imagination: Essays on education, the arts and social change. Jossey Bass.

[bibr19-10778004231172227] Kubota H. Raymond H. Caine V. Clandinin D. J . (2021). Understanding social inclusion: A narrative inquiry into the experiences of refugee families with young children. International Journal of Early Years Education, 30(2), 184–198. 10.1080/09669760.2021.1998885

[bibr20-10778004231172227] Lugones M. (1987). Playfulness, “world”-travelling, and loving perceptions. Hypatia, 2(2), 3–19.

[bibr21-10778004231172227] McKenzie-Robblee S. Steeves P . (2008). Building leadership capacity among student teachers: A narrative inquiry into relational continuity in student teachers’ field placements. LEARNing Landscapes, 1(2), 141–160. 10.36510/learnland.v1i2.260

[bibr22-10778004231172227] Minnich E. (2014). The evil of banality. Arendt revisited. Arts & Humanities in Higher Education, 13(1–2), 158–179.

[bibr23-10778004231172227] Nayeri D. (2019). The ungrateful refugee: What immigrants never tell you. Catapult.

[bibr24-10778004231172227] Noddings N. (1984). Caring. A relational approach to ethics and moral education. University of California Press.

[bibr25-10778004231172227] Noddings N. (2013). Caring. A relational approach to ethics and moral education (3rd ed.). University of California Press.

[bibr26-10778004231172227] Riessman C. (2008). Narrative methods for the human sciences. SAGE.

[bibr27-10778004231172227] Seigfried C. H. (2002). Introduction to the Illinois edition. In Addams J. (Ed.), Democracy and social ethics (pp. ix–xxxviii). University of Illinois Press.

[bibr28-10778004231172227] Steeves P . (2000). Crazy quilt: Continuity, identity and a storied school landscape in transition — A teacher’s and a principal’s works in progress (Unpublished doctoral dissertation). University of Alberta, Edmonton, Alberta, Canada.

[bibr29-10778004231172227] Tubaro P. (2021). Whose results are these anyway? Reciprocity and the ethics of “giving back” after social network research. Social Networks, 57, 65–73. 10.1016/j.socnet.2019.10.003

[bibr30-10778004231172227] Višňovský E. (2011). The pragmatist conception of altruism and reciprocity. Human Affairs, 21(4), 437–453. 10.2478/s13374-011-0042-4

[bibr31-10778004231172227] von Vacano M . (2019). Reciprocity in research relationships: Introduction. In Stodulka T. Dinkelaker S. Thajib F. (Eds.), Affective dimensions of fieldwork and ethnography. theory and history in the human and social sciences (pp. 79–86). Springer.

